# Appendectomy, Tonsillectomy and Parkinson's Disease Risk: A Swedish Register-Based Study

**DOI:** 10.3389/fneur.2020.00510

**Published:** 2020-06-05

**Authors:** Bojing Liu, Fang Fang, Weimin Ye, Karin Wirdefeldt

**Affiliations:** ^1^Department of Medical Epidemiology and Biostatistics, Karolinska Institutet, Stockholm, Sweden; ^2^Institute of Environmental Medicine, Karolinska Institutet, Stockholm, Sweden; ^3^Department of Clinical Neuroscience, Karolinska Institutet, Stockholm, Sweden

**Keywords:** Parkinson's disease, appendectomy, tonsillectomy, nested case-control, register-based

## Abstract

**Introduction:** The gut-brain hypothesis proposes that Parkinson's disease (PD) pathology may start in the gut and later spread to the brain in a prion-like manner. As PD pathology is redundant in the appendix and tonsils, which are important gut-associated lymphoid tissues, we examined whether appendectomy and tonsillectomy were associated with later PD risk.

**Methods:** The nested case-control study included 78,650 PD patients born in 1900–1980 and with a diagnosis of PD between 1964 and 2010. For each PD patient, we randomly selected 40 non-PD controls individually matched for sex and year of birth at the date of PD diagnosis. Appendectomy and tonsillectomy before PD diagnosis were ascertained from the Swedish Patient Register from 1964 onward. We calculated odds ratios (OR) with 95% confidence intervals (CI) using conditional logistic regression adjusting for country of birth, highest achieved education, COPD, comorbidity index, and number of hospital visits.

**Results:** Overall, we found 16% lower risk of PD linked to previous appendectomy (OR = 0.84, 95% CI: 0.80–0.88) and 8% lower risk of PD linked to previous tonsillectomy, although not statistically significant (OR = 0.92, 95% CI: 0.81–1.04). A 7 and 15% lower risk of PD was also noted ≥20 years after appendectomy and tonsillectomy, respectively. Similar associations were observed for men and women but were stronger for PD diagnosed after age 60.

**Conclusion:** Appendectomy and potentially also tonsillectomy were associated with a lower risk PD. A potential mechanism may involve surgical removal of alpha-synuclein redundancy in the appendix and tonsils.

## Introduction

The so-called dual-hit hypothesis about the pathogenesis of idiopathic Parkinson's disease (PD) states that neutrophic pathogens may enter the brain through two portals— the nasal cavity and the gut (1–3). Deposition of alpha-synuclein has been found throughout the entire gut with most dense expression in the appendix of both PD patients and healthy individuals (4). Although still controversial, there is evidence that alpha-synuclein pathology may be transported from cell to cell and thereby spread from the nasal cavity or gut to the brain (2, 3). Mounting evidence has also linked neuroinflammation with PD development (5).

The tonsils and the appendix are important gut-associated lymphoid tissues in the mucosa-associated immune system (6). Surgical procedures to remove these organs often occur in early childhood or adolescence due to recurrent or acute infections, which may cause long-term alteration in immune function. Previous studies have linked these procedures to risk of multiple sclerosis (7) and inflammatory bowel diseases (8). Given the potential spread of alpha-synuclein from the nasal cavity and gut to the brain and the involvement of the tonsils and appendix in immune function, one might hypothesize that surgical removal of these organs may lower the risk of PD. However, previous results are inconclusive, two studies reported that appendectomy was linked to lower risk of PD or delayed PD onset (9, 10), whereas four other studies suggested either a slightly increased PD risk after appendectomy or no association (11–14). Evidence for tonsillectomy and PD risk is scarce; one study reported no association between tonsillectomy and PD risk (15).

We aimed to evaluate the association of appendectomy and tonsillectomy with PD risk in Swedish nationwide registers. In addition to examining appendectomy and tonsillectomy as binary exposures, we also explored the potential temporal relationship between these surgeries and PD risk and the impact of sex and age on the associations.

## Materials and Methods

### Swedish Health and Population Registers

The Swedish *Patient Register* was established in 1964–1965 and collects information on dates of admission and discharge of hospitalizations, surgical procedures, and medical diagnoses (16, 17). Coverage of this register became complete in 1987 and was expanded to cover surgical day-care procedures in 1997 and outpatient visits in 2001 (18). The *Causes of Death Register* records nationwide information on deaths since 1952, and the *Total Population Register* contains information on dates of immigration and emigration (19). The Swedish *Population and Housing Censuses* were conducted every 5 years from 1960 to 1990 to collect detailed information on housing, civil status, and socioeconomic status (20).

### Ascertainment of PD

PD cases were identified from the Patient Register using Swedish revisions of International Classification of Diseases (ICD) codes (i.e., ICD-7: 350 in 1964–1968; ICD-8: 342 in 1969–1986; ICD-9: 332.0 in 1987–1996; and ICD-10: G20 from 1997 onward). Both primary and secondary PD diagnoses were considered. PD is assigned as the primary diagnosis in the Patient Register when PD is considered the main reason for hospitalization, whereas it is assigned as a secondary diagnosis when another condition is considered the main reason. In a previous validation study, compared to clinical workup, positive predicted value (PPV) for inpatient PD diagnosis was 70.8% for primary or secondary PD diagnosis and increased to 80.3% when restricted to primary diagnosis (21).

### Ascertainment of Appendectomy and Tonsillectomy

Information on appendectomy was obtained from the Patient Register according to the Swedish Classification of Operations and Major Procedures codes (4510, 4511, 4517, 0058 in 1964-1996, and JEA00, JEA01, JEA10 from 1997 onward). Information on tonsillectomy was obtained using the codes 2710 and 2720 in 1964-1996, and EMB10, EMB20, EMB30, EMB99 from 1997 onward.

### Study Design

We conducted a nested case-control study based on individuals who were born between 1900 and 1980 and who participated in the Swedish Population and Housing Census in 1970 or 1980. PD cases were identified from the Patient Register between January 1, 1964 and December 31, 2010. For each PD case, we randomly selected 40 controls who were alive and living in Sweden without previous PD diagnosis, individually matched to the PD patient on sex and year of birth on the date of PD diagnosis. Date of PD diagnosis is hereafter referred to as index date. The final study population encompassed 78,650 PD cases and 3,146,000 non-PD controls.

### Co-variates

We retrieved information on country of birth (Swedish vs. non-Swedish born) from the Total Population Register and data on educational attainment (≤9, 10–12, ≥13 years, or unknown) from the Swedish Register of Education. Smoking has consistently been linked to lower risk of PD (22) and also to higher risk of appendectomy (23) and tonsillitis, which is the main indication for tonsillectomy (24). As self-reported information on smoking was not available, we used lifetime chronic obstructive pulmonary disease (COPD) as a proxy for smoking similar to a previous study (25). We obtained information on comorbidity from the Patient Register between 1964 and index date, and further weighted and categorized this information according to Deyo's modification of the Charlson's Comorbidity Index (0, 1–2, or ≥3 points) (26) ICD codes were presented in our previous study (25). As a measurement of surveillance bias related to comorbidity, including appendicitis or tonsillitis, such that these individuals may have more frequent hospital visits and therefore greater likelihood of receiving PD diagnosis compared to others, we obtained information on number of hospital visits before index date from the Patient Register (both inpatient and outpatient), categorized according to tertiles (0–1, 2, or ≥3 visits). Age at index date was categorized as <60, 60–69, 70–79, or >80 years.

### Statistical Analysis

The associations between appendectomy, tonsillectomy, and PD risk were expressed as odds ratios (ORs) with 95% confidence intervals (CIs) estimated from conditional logistic regression. We first analyzed the combined effect of appendectomy and tonsillectomy in relation to PD risk. Individuals who underwent either appendectomy or tonsillectomy or both were defined as exposed and were compared with individuals who had neither surgery regarding PD risk. Second, we performed separate regression analyses for appendectomy and tonsillectomy in relation to PD risk. We performed the above described analyses in two adjustment steps: first, conditional on sex and birth year matched sets; second, additionally adjusted for country of birth, educational attainment, COPD, comorbidity index, and number of hospital visits. We performed temporal relationship analyses exploring PD risk ≥5, ≥10, and ≥20 years after surgeries. We conducted sub-analyses including interaction terms between surgery and sex, as well as surgery and age at index date (<60, 60–69, 70–79, or ≥80). In addition, we performed a sensitivity analysis restricted to PD cases identified through primary diagnosis. We used Stata 15 and SAS 9.4 for statistical analyses with 2-sided alpha of 0.05.

## Results

The distributions of sex and year of birth were balanced between PD cases and controls due to matching (Table 1). The mean age (±SD) at PD diagnosis was 74.0 ± 9.19 years. Individuals who were born outside of Sweden, had ≤ 9 or 10–12 years of education, or had COPD showed lower PD risk. Individuals with unknown educational attainment, more comorbidities, or more frequent hospital visits had a higher risk of PD.

**Table 1:** Characteristics of Parkinson's disease (PD) cases and controls from the Swedish total population 1,964–2,010, *N* = 3,224,650.

	**PD cases, *N* (%)**	**Controls, *N* (%)**	**OR (95% CI)^a^**	***P*-value^b^**
Total	78,650 (100)	3,146,000 (100)		
**Sex**				
Male	43,533 (55.4)	1,741,320 (55.4)		
Female	35,117 (44.6)	1,404,680 (44.6)		
**Age at index date, years**				
<60	5,827 (7.4)	234,176 (7.4)		
60–69	16,041 (20.4)	641,304 (20.4)		
70–79	36,039 (45.8)	1,440,524 (45.8)		
≥80	20,743 (26.4)	829,996 (26.4)		
Born in Sweden				<0.01
Unknown	2 (0)	815 (0)		
No	4,464 (5.7)	186,688 (5.9)	0.95 (0.92–0.98)	
Yes	74,184 (94.3)	2,958,497 (94)	1	
Highest achieved education, years				<0.0001
Unknown	25,126 (31.9)	955,824 (30.4)	1.81 (1.72–1.90)	
≤ 9	30,247 (38.5)	1,273,971 (40.5)	0.89 (0.86–0.91)	
10–12	15,861 (20.2)	640,729 (20.4)	0.92 (0.89–0.95)	
>12	7,416 (9.4)	275,476 (8.8)	1	
Chronic obstructive pulmonary disease (COPD)				<0.0001
No	74,818 (95.1)	2,930,034 (93.1)	1	
Yes	3,832 (4.9)	215,966 (6.9)	0.69 (0.67–0.72)	
Comorbidity index				<0.0001
0	50,235 (63.9)	2,167,220 (68.9)	1	
1–2	21,768 (27.7)	749,584 (23.8)	1.29 (1.27–1.31)	
≥3	6,647 (8.5)	229,196 (7.3)	1.31 (1.27–1.34)	
Number of hospital visits				<0.0001
0–1	24,021 (30.5)	1,310,741 (41.7)	1	
2	20,913 (26.6)	755,964 (24.0)	1.72 (1.69–1.76)	
≥3	33,716 (42.9)	1,079,295 (34.3)	2.19 (2.15–2.24)	

a Logistic regression conditional on birth year and sex;

b *Wald-test p-value for categorical variables*.

### Appendectomy and PD Risk

We identified a total of 80,028 individuals who had an appendectomy. We observed a marginally significant 2% decreased PD risk after an appendectomy after adjusting for sex and birth year matching pairs (Table 2, model 1), and 16% decreased PD risk (HR = 0.84, 95% CI = 0.80–0.88) when additionally adjusted for country of birth, highest achieved education, COPD, comorbidity index, and number of hospital visits (Table 2, model 2). In the temporal relationship analyses, we noted 31, 24, and 22% lower risk of PD with in 5, 10, and 20 years after the surgery, respectively. The inverse associations remained statistically significant with 13, 12, and 7% decreased PD risk observed more than 5, 10, or 20 years post the surgery (Table 2, model 2). We found similar results for men and women and slightly stronger associations for PD diagnosed after age 60 (Table 2, model 2). Similar results were observed when restricted to PD defined through primary diagnosis (Table S1).

**Table 2:** Appendectomy and risk of Parkinson's disease (PD), nationwide case-control analysis.

***N***	**PD cases**	**Controls**	**Model 1^a^^a^**	**Model 2^b^**
	***N***	**OR (95% CI)**	**OR (95% CI)^b^**	
**Appendectomy**				
No	76,742 (97.6)	3,067,880 (97.5)	1	1
Yes	1,908 (2.4)	78,120 (2.5)	0.98 (0.93–1.02)	0.84 (0.80–0.88)
**Years before index date**				
<5	232 (0.3)	11,104 (0.4)	0.84 (0.73–0.95)	0.69 (0.60–0.79)
≥5	1,676 (2.1)	67,016 (2.1)	1.00 (0.95–1.05)	0.87 (0.83–0.92)
<10	511 (0.6)	22,371 (0.7)	0.91 (0.84–1.00)	0.76 (0.69–0.83)
≥10	1,397 (1.8)	55,749 (1.8)	1.00 (0.95–1.06)	0.88 (0.83–0.93)
<20	1,025 (1.3)	44,027 (1.4)	0.93 (0.87–0.99)	0.78 (0.73–0.83)
≥20	883 (1.1)	34,093 (1.1)	1.04 (0.97–1.11)	0.93 (0.87–0.99)
**Stratified by Sex**				
Male	874 (1.1)	36,133 (1.1)	0.97 (0.90–1.03)	0.84 (0.78–0.90)
Female	1,034 (1.3)	41,987 (1.3)	0.98 (0.92–1.05)	0.85 (0.80–0.90)
**Stratified by age at index date, years**				
<60	253 (0.3)	9,538 (0.3)	1.06 (0.94–1.21)	0.90 (0.79–1.02)
60–69	444 (0.6)	17,756 (0.6)	1.00 (0.91–1.10)	0.85 (0.77–0.93)
70–79	776 (1.0)	32,535 (1.0)	0.95 (0.89–1.02)	0.82 (0.76–0.88)
≥80	435 (0.6)	18,291 (0.6)	0.95 (0.86–1.05)	0.86 (0.78–0.94)

a Logistic regression conditional on birth year and sex;

b *Logistic regression conditional on birth year and sex, and additionally adjusted for country of birth, highest achieved education, COPD, comorbidity index, and number of hospital visits*.

### Tonsillectomy and PD Risk

We identified 9,341 individuals who underwent tonsillectomy. There was a trend that previous tonsillectomy was associated with a lower risk of PD in the fully adjusted model (Table 3, model 2), with a more prominent a risk reduction for tonsillectomy performed more than 20 years before PD diagnosis, but the associations were not statistically significant (Table 3, model 2). Similar results were observed when restricted to PD defined through primary diagnosis (Table S2).

**Table 3:** Tonsillectomy and risk of Parkinson's disease (PD), nationwide case-control analysis.

***N***	**PD cases**	**Controls**	**Model 1^a^^a^**	**Model 2^b^**
	***N***	**OR (95% CI)**	**OR (95% CI)^b^**	
**Tonsillectomy**				
No	78,409 (99.7)	3,136,900 (99.7)	1	1
Yes	241 (0.3)	9,100 (0.3)	1.06 (0.93–1.21)	0.92 (0.81–1.04)
**Years before index date**				
<5	26 (0)	954 (0)	1.09 (0.74–1.61)	0.91 (0.62–1.34)
≥5	215 (0.3)	8,146 (0.3)	1.06 (0.92–1.21)	0.92 (0.80–1.05)
<10	59 (0.1)	2,014 (0.1)	1.17 (0.90–1.52)	0.98 (0.75–1.27)
≥10	182 (0.2)	7,086 (0.2)	1.03 (0.89–1.19)	0.90 (0.77–1.04)
<20	136 (0.2)	4,699 (0.1)	1.16 (0.98–1.37)	0.98 (0.83–1.16)
≥20	105 (0.1)	4,401 (0.1)	0.95 (0.79–1.16)	0.85 (0.70–1.03)
**Stratified by Sex**				
Male	141 (0.2)	5,247 (0.2)	1.08 (0.91–1.27)	0.92 (0.78–1.09)
Female	100 (0.1)	3,853 (0.1)	1.04 (0.85–1.27)	0.91 (0.75–1.11)
**Stratified by age at index date, years**				
<60	93 (0.1)	3,081 (0.1)	1.21 (0.98–1.50)	1.02 (0.83–1.26)
60–69	63 (0.1)	2,600 (0.1)	0.97 (0.75–1.25)	0.83 (0.64–1.07)
70–79	62 (0.1)	2,489 (0.1)	1.00 (0.77–1.28)	0.88 (0.68–1.13)
≥80	23 (0)	930 (0)	0.99 (0.65–1.50)	0.90 (0.60–1.36)

a Logistic regression conditional on birth year and sex;

b *Logistic regression conditional on birth year and sex, and additionally adjusted for country of birth, highest achieved education, COPD, comorbidity index, and number of hospital visits*.

## Discussion

In this nationwide nested case-control study, we found a lower PD risk in relation to appendectomy and a non-significant trend toward lower PD risk in relation to tonsillectomy. The inverse associations were generally stronger within 20 years after surgery but remained statistically significant more than 20 years post-surgery. The associations were similar in men and women, but stronger after age 60 compared to before.

According to Braak's hypothesis, alpha-synuclein, the hallmark for PD pathology, may originate in the gut and later migrate to the brain via the vagus nerve (1). In line with this, deposits of alpha-synuclein have been observed in the entire gastrointestinal tract more than 20 years before PD onset (27). Notably, however, out of the entire gastrointestinal system, mucosal alpha-synuclein was most abundant in the appendix in individuals without neurological disease (28). Alpha-synuclein aggregates were equally abundant in normal and inflamed appendiceal tissue (10). More intriguingly, we and others reported lower PD risk more than 20 years after vagotomy (i.e., a surgical procedure resecting the vagus nerve) (25, 29). This evidence collectively suggests that the appendix may act as a reservoir for alpha-synuclein, and, in line with our results, removing the appendix may be linked to lower risk for PD.

Braak's hypothesis was further extended to the dual-hit hypothesis stating that environmental neurotrophic pathogens may spread to the brain from the nasal gateway as well as from the gut (2, 3). Moreover, there is an increasing recognition of PD as a “prion-like” disease supported by the observations of cell-to-cell α-synuclein transmission in grafted neurons in PD patients (30, 31) and the spread of intragastrical injected α-synuclein from the enteric nerve system to the brain in mice (32). The tonsils and the appendix are important gut-associated lymphoid tissues that together with mesenteric lymph nodes, protect hosts against gastro-intestinal infections (6). In transmissible prion-disease, such as Creutzfeld Jakob disease, prions first accumulate within gut-associated lymphoid tissues such as tonsils and the appendix, and later spread to the brain via the enteric nervous system (6). A similar pattern may be hypothesized for the spreading of alpha-synuclein in PD.

One potential explanation for our observed decreased PD risk related to appendectomy is bias. Residual confounding is one potential bias that needs to be considered. Cigarette smoking has been consistently linked to lower risk of PD (22), and although evidence is scarce, it has also been associated with higher risk of appendicitis (23) and chronic or recurrent tonsillitis (24). As this was a register-based study, we unfortunately lacked information on cigarette smoking, but we adjusted for COPD as a proxy for smoking. Another potential bias is reverse causation, which can be present if PD patients would be less likely to be diagnosed with or undergo appendectomy compared to the general population, for example due to very advanced PD. Further, PD has a long prodromal phase (33) and there is a delay between onset of PD symptoms to first inpatient register diagnosis of about 7 years (21). Taking advantage of the long follow-up time in our study, we performed temporal analyses that addressed this issue. Even though we found a stronger inverse association during the period shortly before appendectomy compared to the period longer before, we still observed a statistically significant inverse association more than 20 years before appendectomy. If the hypothesis that appendectomy or tonsillectomy protects against PD is true, a dose-response effect might be expected, such that the inverse association would be stronger with longer duration between surgery and PD. We may speculate that one reason that we did not observe such pattern is reverse causation, but we find it unlikely that our results are explained entirely by reverse causation. Another potential bias is incomplete coverage of the Patient Register before it became nationwide. Individuals who lived in counties covered by the register may be more likely to have both surgery and PD diagnoses captured and vice versa, but this would introduce an underestimation of an inverse association, meaning that the true association would be even stronger than the one observed.

Our results are in line with the previous studies that reported lower PD risk after appendectomy (9, 10). The Killinger et al. study (10) was also based on Swedish register data, but we used a different study design and different definitions of appendectomy and PD, resulting in that our study identified more than 3 times as many PD cases with appendectomy. We also adjusted for several co-variates, including COPD as a proxy for smoking, which they did not. Three previous studies reported that appendectomy was not related to PD risk (11, 13, 14) and one reported higher PD risk after appendectomy (12). Potential explanations include differential definitions of PD cases and controls, surveillance bias, which may result in an artificial positive association between PD and surgery, and inadequate length of follow-up precluding exploration of a potential long-term protective effect of appendectomy on PD. Our results for tonsillectomy were consistent with the Danish study that reported 5% non-significant lower risk of PD after tonsillectomy.

The main strengths of this study are the large population-based sample of more than 3.2 million individuals and the long study period between 1964 and 2010, which allowed us to examine the time-dependent relationship between appendectomy, tonsillectomy, and risk of PD. We used a rigorous matching design and adjusted for several covariates to reduce confounding. We performed sensitivity analysis restricted to primary PD diagnosis to test the robustness of the results. The study also has some limitations. First, PD register diagnoses are not perfect and there is a delay between onset of motor symptoms and first inpatient register PD diagnosis (21). However, the potential misclassification of PD is likely to be non-differential with regard to appendectomy or tonsillectomy, which would result in diluted estimates of the associations. Second, due to lack of information in the registers we were not able to control for potential confounding by lifestyle factors and medication use.

In conclusion, our data suggest that appendectomy and potentially also tonsillectomy are associated with a decreased risk of PD. A potential mechanism may be the surgical removal of alpha-synuclein redundancy in these organs. Our data provide additional evidence supporting the importance of the gut-to-brain axis in PD etiology.

## Data Availability Statement

The data analyzed in this study were obtained from the Swedish National Board of Health and Welfare and Statistics Sweden and because of Swedish privacy laws we cannot make the data publicly available. Requests to access these datasets should be directed to the Swedish National Board of Health and Welfare and Statistics Sweden after obtaining an ethical approval from a regional ethics review board.

## Ethics Statement

The studies involving human participants were reviewed and approved by the Regional Ethics Review Board, Stockholm, Sweden. Written informed consent for participation was not required for this study in accordance with the national legislation and the institutional requirements.

## Author Contributions

KW and FF were responsible for study concept, study design and funding. BL performed data management and statistical analysis as well as drafted the manuscript. All authors contributed to interpretation of results and critical revision of the manuscript.

## Conflict of Interest

The authors declare that the research was conducted in the absence of any commercial or financial relationships that could be construed as a potential conflict of interest.
