# Supplementary material for: Appendectomy, Tonsillectomy and Parkinson's Disease Risk: A Swedish Register-Based Study
Source: Front Neurol. 2020 Jun 5;11:510. doi: 10.3389/fneur.2020.00510 (PMC7292857; doi:10.3389/fneur.2020.00510)
Supplement: Supplementary file 1 [file Table_1.DOCX]

Supplementary Material

# Supplementary Figures and Tables

**eTable 1** Appendectomy and risk of Parkinson disease (PD) in nationwide case-control study: sensitivity analyses restricting to primary PD register diagnosis

|  | **PD/control ^a^** | **OR (95% CI)^a^** |
| --- | --- | --- |
| **Appendectomy** | | |
| No | 38,773/1,548,884 | 1 |
| Yes | 1,078/45,156 | 0.87 (0.82-0.93) |
| Years before index date | |  |
| <5 | 102/5,812 | 0.63 (0.52-0.76) |
| ≥5 | 976/39,344 | 0.91 (0.85-0.97) |
| <10 | 247/11,849 | 0.75 (0.66-0.85) |
| ≥10 | 831/33,307 | 0.92 (0.85-0.98) |
| <20 | 534/23,853 | 0.81 (0.74-0.88) |
| ≥20 | 544/21,303 | 0.95 (0.87-1.03) |
| **Stratified by Sex** | |  |
| Male | 485/20,639 | 0.86 (0.78-0.94) |
| Female | 593/24,517 | 0.88 (0.81-0.96) |
| **Stratified by age at index date, years** | | |
| <60 | 203/7,942 | 0.91 (0.79-1.05) |
| 60-69 | 307/12,523 | 0.89 (0.79-0.99) |
| 70-79 | 407/17,809 | 0.84 (0.76-0.93) |
| ≥80 | 161/6,882 | 0.89 (0.76-1.04) |

a. restricted to *primary PD cases* and controls, logistic regression conditional on sex and birth year matching pairs, additionally adjusted for country of birth, highest achieved education, COPD, comorbidity index, and number of hospital visits.

**eTable 2** Tonsillectomy and risk of Parkinson disease (PD) in nationwide nested case-control study: sensitivity analyses restricting to primary PD register diagnosis

|  | **PD/control ^a^** | **OR (95% CI)^a^** |
| --- | --- | --- |
| **Tonsillectomy** | | |
| No | 39,679/1,587,500 | 1 |
| Yes | 172/6,540 | 0.96 (0.82-1.11) |
| Years before index date | |  |
| <5 | 16/596 | 0.99 (0.60-1.62) |
| ≥5 | 156/5,944 | 0.95 (0.81-1.12) |
| <10 | 34/1,275 | 0.96 (0.68-1.35) |
| ≥10 | 138/5,265 | 0.95 (0.81-1.13) |
| <20 | 89/3,188 | 1.00 (0.81-1.24) |
| ≥20 | 83/3,352 | 0.91 (0.73-1.13) |
| **Stratified by Sex** | |  |
| Male | 105/3,767 | 1.00 (0.83-1.22) |
| Female | 67/2,773 | 0.89 (0.70-1.14) |
| **Stratified by age at index date, years** | | |
| <60 | 82/2,647 | 1.11 (0.88-1.38) |
| 60-69 | 48/1,988 | 0.87 (0.65-1.16) |
| 70-79 | 33/1,522 | 0.82 (0.58-1.16) |
| ≥80 | 9/383 | 0.91 (0.47-1.77) |

a. restricted to *primary PD cases* and controls, logistic regression conditional on sex and birth year matching pairs, additionally adjusted for country of birth, highest achieved education, COPD, comorbidity index, and number of hospital visits.
